# A Comparative Study of Early Versus Conventional Initiation of Oral Fluid Intake After the Caesarean Section

**DOI:** 10.7759/cureus.78670

**Published:** 2025-02-07

**Authors:** Chava Kavya, Srividya Kunamneni, Namballa Uma

**Affiliations:** 1 Department of Obstetrics and Gynaecology, NRI Institute of Medical Sciences, Visakhapatnam, IND

**Keywords:** caesarean section, conventional oral feeding, early oral feeding, maternal satisfaction, postoperative ileus

## Abstract

Background: Caesarean delivery is an abdominal surgery and hydration and nutrition during postoperative care is a main concern for women. It is customary to give oral fluids only after 24 hours following the return of bowel sounds. Early oral fluid intake has been recommended for women after caesarean delivery, which may improve earlier gastrointestinal recovery that can facilitate early discharge from hospital. In this study, the patient recovery is compared between the traditional practice of fluid intake versus early fluid intake.

Aim: To compare the efficacy and side effects of early postoperative oral fluid intake versus delayed or conventional oral fluid intake in patients after caesarean section under regional anaesthesia.

Methods: A comparative observational study was conducted on 100 term pregnant women who underwent an uncomplicated caesarean section under regional anaesthesia at the NRI Institute of Medical Sciences, Visakhapatnam, from August 2023 to January 2024. Women were randomized to early and delayed feeding group after taking informed consent. One set of 50 women in the early-fed group were offered a liquid diet within 6-8 hours after surgery and a soft diet in the next 14-16 hours. In another set, 50 women in the control group were offered conventional fluid intake 16-24 hours after the surgery.

Results: Women in the early-fed group had a lower duration of intravenous fluid (IV) fluid administration. There were significant differences between the two groups in the duration of intravenous fluid administration, lesser time interval for flatus passage, reduced rate of ileus symptoms and length of hospital stay. The early-fed group had a significantly shorter mean postoperative time interval to return of bowel sound, passage of flatus and stools (P<0.0001) and also shorter hospital stay.

Conclusion: Early feeding after an uncomplicated caesarean section reduces the rate of ileus symptoms, duration of intravenous fluid administration and leads to a reduced hospital stay with higher maternal satisfaction rates.

## Introduction

Caesarean section is an abdominal surgery and in the postoperative care period, ensuring adequate hydration and nutrition for the woman is the primary focus. After a caesarean section, it is conventional for the patient to remain nil by mouth for a specific period of time or till the return of bowel sounds or passage of flatus [1]. The global rate of caesarean deliveries has been steadily increasing. Traditionally, patients were kept on “nil per mouth” (fasting) until bowel sounds returned or flatus was passed, a period that ranges from 24 to 48 hours. Following this, clear fluids were introduced, gradually progressing to a soft diet. A regular diet was only allowed once the patient passed flatus or had a bowel movement.

The traditional dietary regimen is being practised by many to prevent the incidence of post-operative ileus and consequent vomiting after a caesarean section [2]. Recently, early oral fluid intake has been recommended as an alternative to the traditional dietary regimen for women following a caesarean delivery. Gastrointestinal recovery following caesarean section may be improved by early oral fluid intake [3]. The evidence suggests that earlier resumption of solid food causes accelerated return of bowel activity and reduced duration of hospital stay with no evidence of higher complication rates [4,5]. Early oral feed after a caesarean section is anticipated to promote early ambulation, reduce costs and enhance overall maternal satisfaction. Early oral feeding helps minimize protein storage depletion, accelerates wound healing and supports faster recovery. Moreover, the economic advantages of early discharge after an uncomplicated caesarean delivery are substantial. Our study aimed to compare the effectiveness and side effects of early postoperative oral fluid intake versus delayed or conventional oral fluid intake in patients who underwent caesarean section under spinal anaesthesia.

## Materials and methods

This was a comparative observational study conducted at the NRI Institute of Medical Sciences, Visakhapatnam, from August 2023 to January 2024 with a sample size of 100 term pregnant women.

Inclusion criteria

The study included term pregnant women who underwent an uncomplicated cesarean section under spinal anaesthesia, with an operative time of 60 minutes or less.

Exclusion criteria

The study excluded cases of severe preeclampsia receiving magnesium sulphate therapy, obstructed labour, impending uterine rupture, chorioamnionitis, placenta previa with postpartum haemorrhage and those with an operative time exceeding 60 minutes for any reason.

Institutional ethical committee clearance for conducting the study was obtained on 8 August 2023 with reference code IEC/NRI/70/2023.

Early-fed group versus delayed feeding group

Informed consent was obtained from women who met the inclusion and exclusion criteria. In this study, 100 women were included and randomly assigned to one of the two groups: the early-fed group and the delayed feeding group. The early-fed group consisted of 50 women who were given a liquid diet in 6-8 hours post-surgery and a soft diet at the next meal (14-16 hours), followed by a regular diet. The delayed feeding group, also comprising 50 women, were not allowed oral intake for the first 16-24 hours after surgery. They were then given a liquid diet, a soft diet and then a regular diet. The age, parity, and gestational age of the women in both groups were recorded.

The outcomes following early oral intake were evaluated based on the following criteria: The day of surgery is considered as postoperative day 0 (the first 24 hours). The operating time is taken as the period from the start of surgery to skin closure. The duration of intravenous fluid administration is measured from the onset of surgery to the discontinuation of intravenous (IV) fluids. The first appearance of bowel sounds is used as an indicator for the onset of bowel movement after surgery. Mild ileus symptoms considered were anorexia, abdominal pain, nausea, vomiting and mild abdominal distension on examination. Severe ileus symptoms involve significant abdominal distension, vomiting and the requirement of a nasogastric tube. The duration of hospital stay is calculated from the day of surgery until the patient's discharge. Data were analysed using an independent t-test, and IBM SPSS for Statistics version 24 (IBM Corp, Armonk, NY) was used for statistical analysis.

## Results

In this study, 50 women were included in the early-fed group, where they were given a liquid diet 6-8 hours post-surgery and a soft diet at the next meal (14-16 hours later) followed by a regular diet. The control group also had 50 women, who were not given any oral feeds for the initial 16-24 hours post-surgery, after which they were given a liquid diet, then a soft diet, before transitioning to regular diet.

Table 1 shows the age distribution of both the study and control groups. In the early-fed group, 27 (54%) women were aged 18-25 years, while 23 (46%) were aged 25-40 years. In the delayed feeding group, 31 (62%) were in the 18-25 age range, and 19 (38%) were aged between 25 and 40 years. The age group distribution is similar in both groups.

**Table 1:** Age distribution

Age (years)	Early-fed group (N=50)	Delayed feeding group (N=50)
18-25	27 (54%)	31 (62%)
25-40	23 (46%)	19 (38%)

Table 2 shows the parity distribution in both study and control groups. In this study, among the 50 women in the early-fed group, 20 were primigravida and 25 were multigravida. In the delayed feeding group, 30 women were primigravida and 25 were multigravida. The distribution of parity is similar in both groups.

**Table 2:** Parity distribution

Parity	Early-fed group (N=50)	Delayed feeding group (N=50)
Primigravida	20 (40%)	30 (60%)
Multigravida	25 (50%)	25 (50%)

Table 3 shows the distribution of study and control groups according to the gestational age. In this study, three women (6%) in the early-fed group had a gestational age <37 weeks and 47 (94%) had a gestational age >37 weeks. In the delayed feeding group, seven (14%) had a gestational age <37 weeks and 43 (86%) had a gestational age >37 weeks. Gestational age distribution was found to be similar in both study and control groups.

**Table 3:** Gestational age distribution

Gestational age (in weeks)	Early feeding group	Delayed feeding group
<37 weeks	3 (6%)	7 (14%)
>37 weeks	47 (94%)	43 (86%)

Table 4 shows the time interval to start liquid and soft diets. The early-fed group were allowed a liquid diet after an average of 6.44±0.76 hours, while the delayed feeding group started after 11.68±1.51 hours. The early-fed group received a soft diet after 13.42±1.86 hours, compared to 24.62±2.45 hours in the delayed feeding group with P<0.0001.

**Table 4:** Time interval to the start of diet

Variables	Early feeding group (N=50)	Delayed feeding group (N=50)	p value
Time interval to start liquid diet (hours)	6.44±0.76	11.68±1.51	<0.0001
Time interval to start soft diet (hours)	13.42±1.86	24.62±2.45	<0.0001

Table 5 shows the grade of post-operative ileus in both early and delayed feeding groups. No patients in the early-fed group had ileus and 2% of conventional feeding group had mild symptoms of ileus. The symptoms resolved spontaneously in all patients who had mild ileus.

**Table 5:** Grade of post-operative ileus

Ileus	Early feeding group	Delayed feeding group
Mild	0%	1 (2%)
Severe	0%	0%

Table 6 shows the duration of administration of intravenous fluids in both groups. In our study, women in early fed group had lesser mean duration of administration of intravenous fluid of 15.94+/-1.81 hours and a mean of 31.8+/- 2.74 hours in delayed feeding group, which is statistically significant with p value <0.0001. The mean duration of administration of intravenous fluids was less in early fed group due to early initiation of oral fluids when compared to delayed feeding group.

**Table 6:** Duration of the administration of intravenous fluids

Variables	Group	Mean	Std. deviation	p-value
Duration of IV fluids (hours)	Early feeding group (N=50)	15.94	1.81	<0.0001
	Delayed feeding group (N=50)	31.80	2.74	

Table 7 depicts the time interval for the passage of flatus. In our study, women in the early-fed group had a shorter time interval for the passage of flatus (mean 12.4±1.4 hours) compared to the delayed feeding group (mean 16.92 hours±1.62 hours) with p value of <0.0001, which is statistically significant.

**Table 7:** Time interval for the passage of flatus

Variables	Group	Mean	Std. deviation	p-value
Time interval for passage of flatus (hours)	Early feeding group (N=50)	12.40	1.40	<0.0001
	Delayed feeding group (N=50)	16.92	1.62	

Table 8 shows the time interval for the passage of stools in both early and delayed feeding groups. In our study, the mean time interval for the passage of stools post-surgery in women in the early-fed group was 2.36±0.48 days compared to a mean of 3.36±0.48 days in the delayed feeding group, which is statistically significant (p<0.0001).

**Table 8:** Time interval for the passage of stools

Variables	Group	Mean	Std. deviation	p-value
Time interval for passage of stools (post-operative day)	Early feeding group (N=50)	2.36	0.48	<0.0001
	Delayed feeding group (N=50)	3.36	0.48	

Table 9 depicts the length of hospital stay. The mean duration of hospital stay in the early-fed group was 4.28±0.60 days compared to a mean of 6.78±0.76 days in the delayed feeding group. The length of hospital stay in two groups was found to be statistically significant with a p-value of <0.0001 in our study.

**Table 9:** Length of hospital stay

Variables	Group	Mean	Std. deviation	p-value
Hospital stay (hours)	Early feeding group (N=50)	4.28	0.60	<0.0001
	Delayed feeding group (N=50)	6.78	0.76	

Table 10 presents a comprehensive comparison of outcomes between the early and delayed feeding groups, including the mean values and the corresponding p-value. Women who were given early oral fluids after the caesarean section had a lower duration of the administration of intravenous fluids, lesser time interval for flatus passage, reduced incidence of postoperative ileus and lesser duration of hospital stay. In the control group, one patient (2%) had post-operative ileus symptoms and there is an increased duration of administration of intravenous fluids and increased hospital stay post-operatively.

**Table 10:** Comparison of early versus delayed feeding groups

Variables	Early feeding group	Delayed feeding group	
	Mean	Mean	p-value
Intravenous fluids	15.94±1.81	31.80±2.74	<0.0001
Time interval for the passage of flatus	12.40±1.40	16.92±1.62	<0.0001
Time interval for the passage of stools	2.36±0.48	3.36±0.48	<0.0001
Length of hospital stay	4.28±0.60	6.78±0.76	<0.0001

## Discussion

In this study, we compared the outcomes of early versus traditional oral feeding following caesarean sections done under spinal anaesthesia. Obstetricians have historically delayed oral intake in the post-operative period until the resolution of post-operative ileus, indicated by the passage of flatus. However, recent studies have shown that early oral feeding is both well-tolerated and advantageous for patients who underwent a caesarean section. The early-fed group experienced an early development of bowel sounds, passage of flatus and bowel movements post-surgery. Early feeding is believed to positively impact the gastrointestinal tract by stimulating bowel peristalsis and expedite an earlier return to bowel function.

Gestational age distribution

In our study, among the women in the early-fed group, 6% of the participants had less than 37 weeks of gestation, while 94% had more than 37 weeks of gestation. In the delayed feeding group, 14% had less than 37 weeks and 86% had more than 37 weeks of gestation. Nantasupha et al. reported a mean gestational age of 38.6±0.6 weeks for the early-fed group and 38.6±0.7 weeks for the delayed oral feeding group, based on dates and earlier ultrasound scans [6]. Similarly, in a study by Mehta et al., the mean gestational ages were 38.7±1.3 weeks and 38.5±1.1 weeks by dates and earlier ultrasound scans, respectively, in both groups, yielding comparable results [7].

Timing of initiation of fluid intake

Jalilian et al. evaluated the safety and efficacy of early oral feeding after cesarean delivery under general and regional anesthesia by a randomized controlled trial in 200 women [8]. In their trial, women in the early-fed group were motivated to take sips of water 8 hours after the surgery, followed by 100 ml oral tea under supervision. Women in the routine feeding group were restricted oral intake for the first 24 hours and given sips of water 24-48 hours post-operatively. The incidence of symptoms of paralytic ileus was not significantly different between the two groups (15% vs 13%). In our study, 100 women who underwent uncomplicated caesarean section under spinal anaesthesia were considered. Out of these 100 women, 50 women in the early-fed group were allowed oral fluids 6-8 hours after surgery and a soft diet after 14-16 hours followed by a regular diet. The remaining 50 women in the delayed feeding group were allowed orals 16-24 hours post-surgery followed by a soft diet and regular diet. The incidence of symptoms of paralytic ileus was statistically significant between both groups in our study.

Duration of IV fluids

In a study conducted by Nantasupha et al., the amount of IV fluids administered was 1.1±0.3 litres for women in the early-fed group and 2.8±0.5 litres for the late feeding group, which was statistically significant [6]. Additionally, another study by Kathpalia found that the IV fluid amounts were 4.2±1.2 bottles per patient in the early-fed group and 6.1±0.8 bottles in the late feeding group, a significant difference between both groups [9]. In our study, as we compared the duration of administration of intravenous fluids, a statistically significant difference was found between early and delayed feeding groups (15.94 hours vs 31.8 hours, p<0.0001).

Time interval for the return of bowel sounds

A systematic review and meta-analysis by Hsu et al. also showed a significant reduction in the time to return of bowel sounds in the early oral intake group but did not have a significant effect on post-operative vomiting [10]. In our study, women with an early initiation of oral fluid intake had demonstrated a rapid return of bowel function after a caesarean section with a significant shorter mean post-operative time interval for the passage of flatus. Women in the early-fed group had a shorter time interval for the passage of flatus (mean 12.4±1.4 hours) compared to the delayed feeding group (mean 16.92±1.62 hours) with p-value of <0.0001, which is statistically significant.

Barat and his associates found in a study that for the early oral feeding group, the mean time for the passage of flatus was 10.2±1.7 hours while it was 10.7±1.6 hours in the delayed feeding group, which was statistically significant [11]. In our study, women in early-fed group had a shorter time to the passage of flatus, with a mean of 12.4±1.4 hours, compared to the delayed feeding group, which had a mean of 16.92±1.62 hours. The difference between two groups was statistically significant, with a p-value of <0.0001.

Devi et al. found in another study that women in early-fed group had significantly shorter time interval to first noticed bowel movement after surgery of 6.97±0.71 hours compared to conventionally fed women with 14.96±4.97 hours [1]. In our study, women in the early-fed group had shorter time to passage of stools with a mean of 2.36±0.48 days, compared to 3.36±0.48 days in delayed feeding group. The difference between both groups was found to be statistically significant (p-value < 0.0001).

Sahar and her colleagues defined their study group as participants who received oral hydration after 2 hours post-operatively and the control group as those who initiated oral hydration 8 hours post-operatively and mentioned that the women in the early oral group had more faster return of bowel sounds than the delayed group [12]. In our study, the early-fed group received a liquid diet at an average of 6.44 hours (±0.76 hours) post-surgery, while the delayed feeding group started at 11.68 hours (±1.51 hours). Additionally, the early-fed group was given a soft diet at 13.42 hours (±1.86 hours), compared to 24.62 hours (±2.45 hours) in the delayed feeding group. Furthermore, women in the early-fed group had significantly shorter time to pass flatus, averaging 12.4 hours (±1.4 hours), compared to 16.92 hours (±1.62 hours) in the delayed feeding group, with a statistically significant p-value of less than 0.0001.

Rate of symptoms of paralytic ileus

In a study by Chantarasorn et al., the rate of mild ileus symptoms in the early-fed group was significantly less than the conventional group (19.6% versus 31.1%, p<0.03) [13]. In our study, none of the participants in the early-fed group experienced ileus symptoms, while 2% of women in the conventional feeding group reported mild ileus symptoms, a difference that was statistically significant.

Patolia et al. compared the post-operative outcomes associated with early oral feeding (liquid diet 2 hours after surgery) versus late oral feeding (liquid diet 8 hours after surgery) in 140 women undergoing elective C-section under regional anaesthesia [14]. There were no significant differences between the two groups in the post-operative gastrointestinal complications. In our study, postoperative ileus observed in the delayed feeding group is 2%, whereas there were no ileus symptoms noted in the early-fed group.

Craciunas et al., in his study of 192 women who underwent C-section under both regional and general anaesthesia, observed the effect of the time of start of oral feeding on patient acceptability and also the benefits on gastrointestinal functions [15]. No significant difference in the incidence of paralytic ileus symptoms was found among the early and conventional feeding groups (15.6% versus 29.5%). None of the patients in the early-fed group had ileus symptoms and 2% of the conventional feeding group had mild ileus symptoms in our study.

Adverse gastrointestinal effects

Kovavisarach and Atthakorn observed no significant incidence of adverse gastrointestinal effects after a caesarean section in 151 women who were given early feeds when compared to the women for whom the first meal was delayed (8 hours versus 24 hours) [16]. In our study, we noted mild ileus symptoms in the delayed feeding group and no women in the early-fed group had ileus or adverse gastrointestinal effects.

Duration of hospital stay

A study by Göçmen et al. showed that the time for a sound bowel movement and duration of stay in the hospital was shorter in patients subjected to early feed as compared to late feed [17]. In our study, the duration of hospital stay was significantly different, with the early-fed group averaging 4.28 days (±0.60 days) compared to 6.78 days (±0.76 days) in the delayed feeding group, with a p-value of less than 0.0001. In the study by Ogbadua et al., the duration of hospital stay was 4.2±0.7 days for the early-fed group and 4.2±1.2 days for the late feeding group [5].

Limitations of the study

Our study has some limitations that should be considered when interpreting the results. The exclusion of complicated caesarean sections, such as those involving maternal or fetal complications, may limit the applicability of the findings to women with more complex clinical situations. Women undergoing uncomplicated caesarean sections may recover differently than those with additional medical challenges, so the results may not fully reflect the experiences of all women who have a caesarean delivery. The traditional beliefs and cultural practices surrounding post-operative care may have influenced patient management, particularly in regions where specific customs dictate how and when oral intake is introduced after surgery. As a result, the influence of these beliefs on the study's findings could reduce the generalizability of the results to different populations or settings where these traditions are not as prevalent.

## Conclusions

Early oral fluid intake after a cesarean section has been shown to reduce several key aspects of post-operative recovery. First, it shortens the duration of intravenous (IV) fluid administration, as patients are able to tolerate oral intake sooner, reducing their reliance on IV fluids. This not only decreases the need for a continuous intravenous access but also helps accelerate the transition to a normal diet. Additionally, early oral intake has been associated with a lower incidence of ileus symptoms, which are common after surgery, particularly in abdominal procedures like caesarean sections. Ileus symptoms such as nausea, vomiting, abdominal pain and bloating are typically less severe when patients are allowed to begin drinking fluids sooner. This early intake helps stimulate bowel motility, reducing the risk of prolonged ileus and promoting faster recovery of normal digestive function.

Moreover, early oral fluid intake contributes to a shorter length of hospital stay. By facilitating faster recovery, including the resolution of ileus symptoms and the ability to resume normal nutrition, patients can be safely discharged earlier, which reduces hospital costs and frees up resources for other patients. Lastly, early oral intake has been associated with higher maternal satisfaction. Women often feel more comfortable when they can resume eating and drinking shortly after surgery. This not only enhances their physical recovery but also contributes to a more positive emotional experience during the postpartum period. Being able to start oral intake sooner can improve the overall well-being of women and promote a sense of empowerment, leading to greater satisfaction with their care and recovery process.
